# Catastrophic High-Risk Pulmonary Embolism With Recurrent Cardiac Arrest Successfully Treated With Rescue Mechanical Thrombectomy After Systemic Thrombolysis

**DOI:** 10.7759/cureus.117301

**Published:** 2026-10-01

**Authors:** Vincenzo Cuzzone, Alessia Di Bartolomeo, Nicola Porchetta, Francesco Scarati, Carmen Fierro

**Affiliations:** 1 Anesthesiology and Critical Care, Ospedale Antonio Cardarelli, Campobasso, ITA; 2 Cardiovascular Medicine, Ospedale Antonio Cardarelli, Campobasso, ITA; 3 Internal Medicine, Ospedale Antonio Cardarelli, Campobasso, ITA

**Keywords:** high-risk pulmonary embolism, obstructive shock, recurrent cardiac arrest, rescue mechanical thrombectomy, right ventricular failure, systemic thrombolysis

## Abstract

High-risk pulmonary embolism (PE) complicated by cardiac arrest carries an extremely high mortality, and the optimal rescue strategy after an inadequate response to systemic thrombolysis remains uncertain. We report the case of a 53-year-old woman with previous deep-vein thrombosis who was admitted after an out-of-hospital cardiac arrest and subsequently experienced three additional episodes of cardiac arrest with pulseless electrical activity. Computed tomography pulmonary angiography demonstrated extensive bilateral central PE with severe right ventricular dilation and interventricular septal shift. Systemic thrombolysis with alteplase was administered. Despite thrombolysis and vasopressor/inotropic support, persistent cardiogenic-obstructive shock, electrical instability, and severe right ventricular pressure overload remained. Approximately 4 h after thrombolysis, rescue mechanical aspiration thrombectomy was performed using the Indigo® Aspiration System with Lightning Flash™ (Penumbra, Inc., Alameda, CA, USA). Ultrasound-guided right common femoral venous access was obtained with an 18 F sheath, and aspiration was performed in both pulmonary arteries. Pulmonary artery pressure decreased from 34 mmHg before aspiration to 25 mmHg at the end of the procedure. The patient subsequently achieved progressive hemodynamic stabilization. After prolonged mechanical ventilation and tracheostomy, neurological recovery was favorable. At discharge, echocardiography showed normal biventricular systolic function. This case illustrates that prompt escalation to rescue mechanical thrombectomy may be considered in selected patients with high-risk PE when shock, electrical instability, and right ventricular overload persist despite systemic thrombolysis.

## Introduction

High-risk pulmonary embolism (PE) occurs when a clot obstructing the pulmonary arteries causes cardiac arrest, obstructive shock, or persistent hypotension. Sudden obstruction raises the pressure faced by the right ventricle (RV), which can dilate and pump less effectively. A markedly enlarged RV can shift the interventricular septum toward the left ventricle, impair its filling, and reduce systemic blood flow. Cardiac arrest may present as pulseless electrical activity (PEA), in which organized electrical activity is present without an effective pulse. Mortality is particularly high when cardiopulmonary resuscitation (CPR) is required [1,2].

Systemic thrombolysis is the established first-line reperfusion strategy for hemodynamically unstable PE. When thrombolysis is contraindicated or fails to restore hemodynamic stability, surgical embolectomy or catheter-directed treatment may be considered. Contemporary guidance recognizes mechanical thrombectomy as a reasonable advanced therapy in selected patients with persistent shock, although high-quality comparative evidence remains limited [1,3,4].

We describe recurrent PEA arrest and persistent shock after alteplase in a patient with bilateral high-risk PE. Bilateral aspiration thrombectomy was undertaken approximately four hours after thrombolysis. We distinguish the immediate procedural pressure change from the slower subsequent clinical recovery.

## Case presentation

Urgent computed tomography pulmonary angiography showed extensive bilateral central pulmonary arterial thrombi with near-complete obstruction of the main branches, marked right ventricular dilation, and leftward septal shift (Figure 1). Available bedside transthoracic echocardiographic parasternal long-axis images also showed RV enlargement relative to the left ventricle (Figure 2). Apical four-chamber images, quantitative right ventricular function, and an echocardiographic estimate of pulmonary artery systolic pressure cannot be retrieved from the clinical record; the extent of right ventricular dysfunction should therefore be interpreted with this limitation. Metabolic acidosis and elevated lactate were described clinically as markers of impaired perfusion, but reliable contemporaneous numeric values cannot be retrieved from the clinical record.

**Figure 1:**
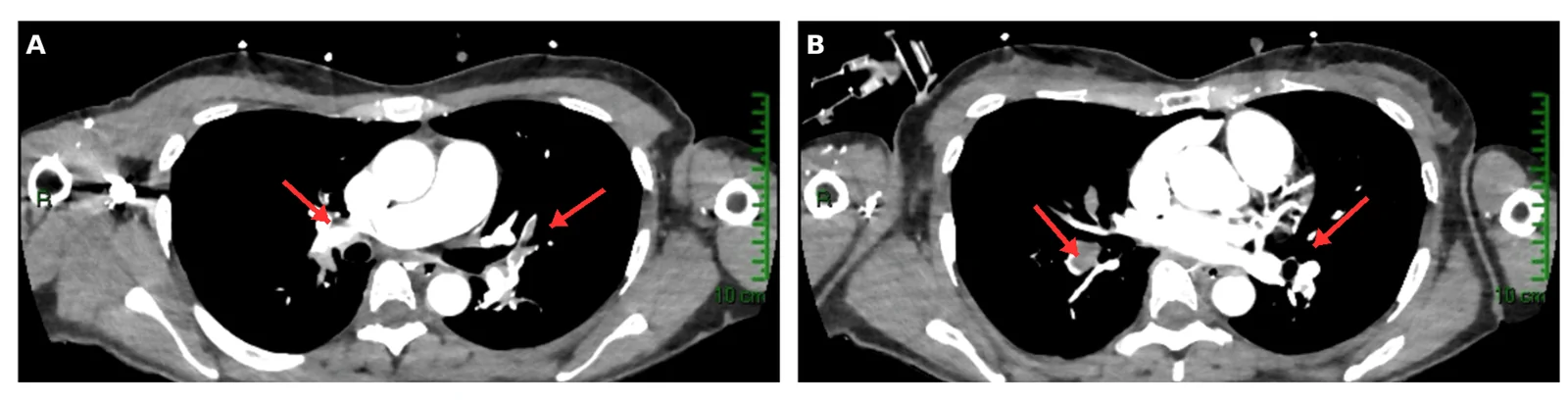
Computed tomography pulmonary angiography at presentation. Axial images (A and B) show extensive bilateral central filling defects involving the right and left pulmonary arterial branches (red arrows).

**Figure 2:**
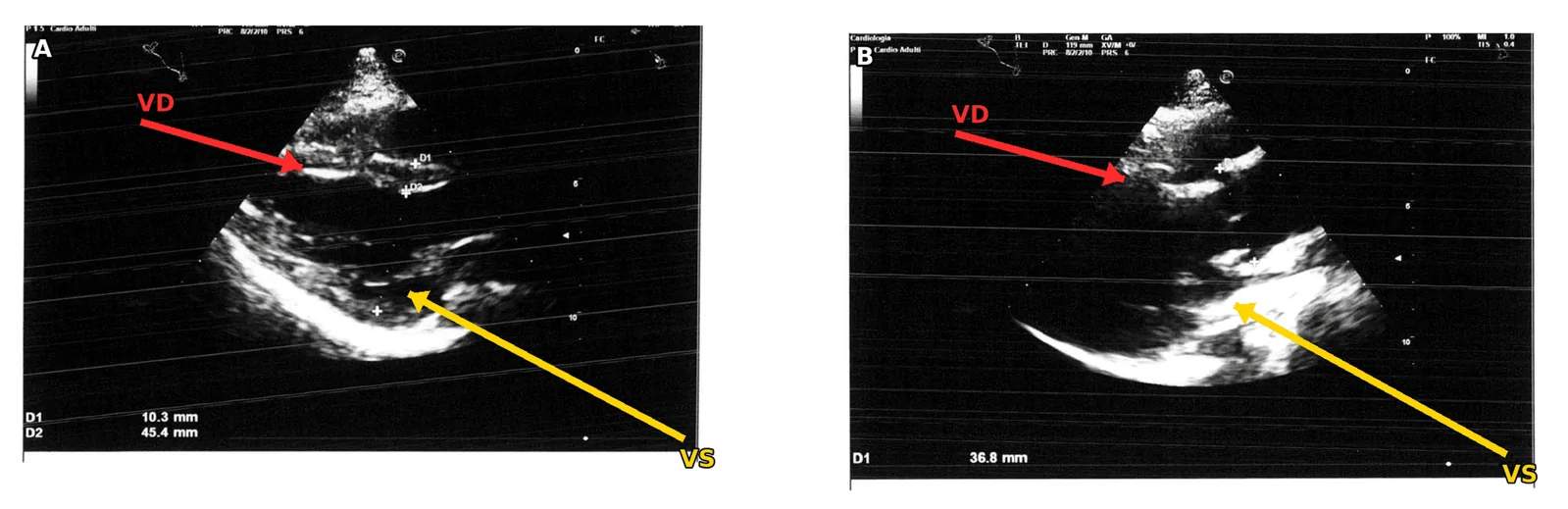
Available bedside transthoracic echocardiographic parasternal long-axis images at presentation (A and B). These views show apparent right ventricular enlargement. RV (labeled VD in the original images, red arrows), right ventricle; LV (labeled VS, yellow arrows), left ventricle. Apical four-chamber and quantitative RV measurements cannot be retrieved from the clinical record.

Approximately 4 h after systemic thrombolysis, the inadequate clinical response prompted emergency rescue mechanical aspiration thrombectomy with the Indigo® Aspiration System with Lightning Flash™ (Penumbra, Inc., Alameda, CA, USA). Ultrasound-guided right common femoral venous access was obtained, and an 18 F introducer sheath was placed. Bilateral aspiration thrombectomy of the right and left pulmonary arteries retrieved substantial thrombotic material (Figure 3).

**Figure 3:**
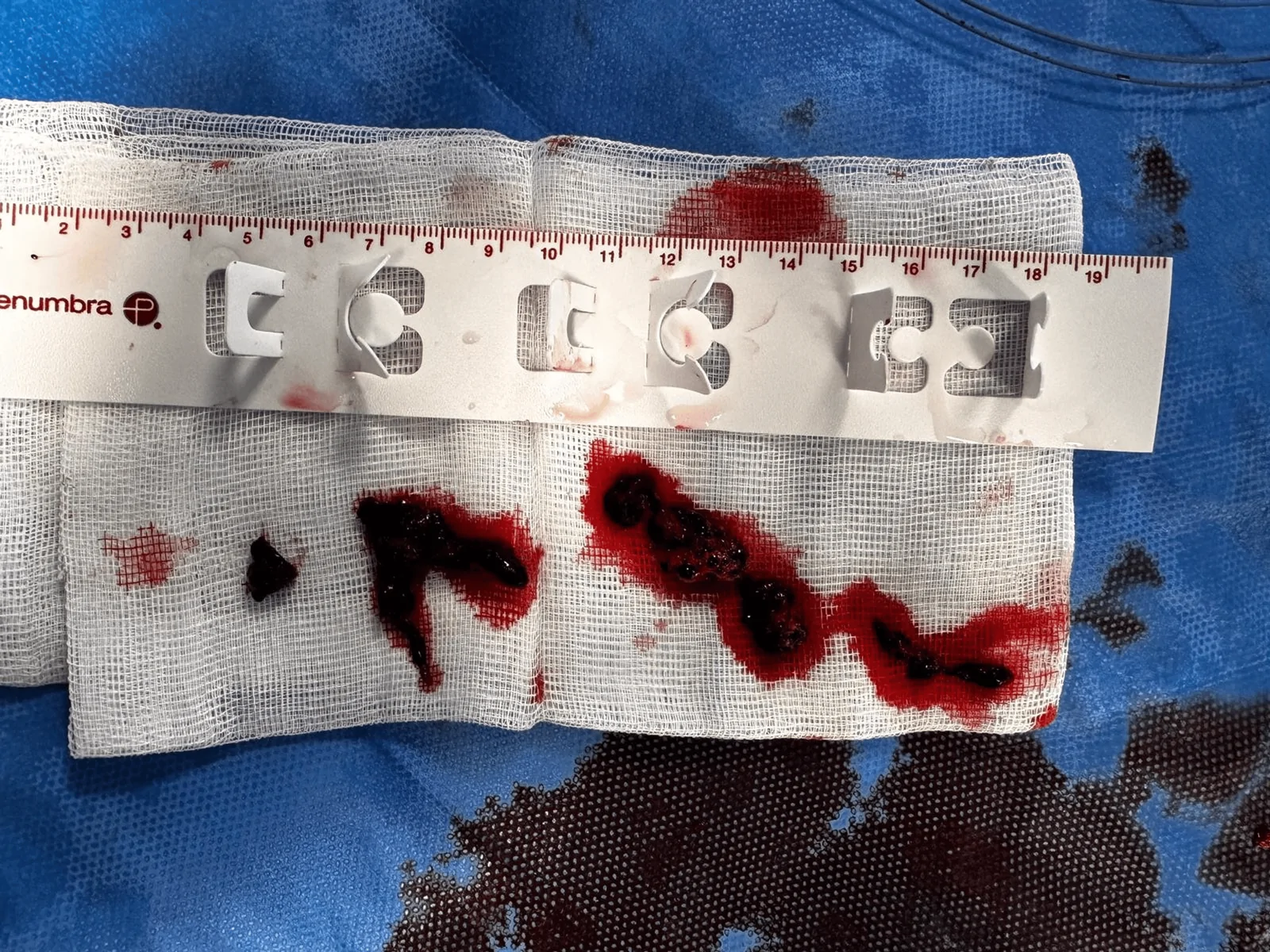
Thrombotic material retrieved during bilateral pulmonary artery aspiration thrombectomy with the Indigo® Aspiration System with Lightning Flash™. Indigo® Aspiration System with Lightning Flash™: Penumbra, Inc., Alameda, CA, USA.

Recorded pulmonary artery pressure decreased from 34 mmHg before aspiration to 25 mmHg at the end of the procedure. The source does not specify whether this was mean, systolic, or another pulmonary artery pressure measure; we therefore report it only as recorded. Continuous intravenous unfractionated heparin was administered after thrombectomy with clinical and laboratory monitoring. The immediate pressure decrease was a procedural observation and did not constitute immediate resolution of shock.

Given the severity of the thromboembolic event, an extended thrombophilia work-up was performed. Hematological evaluation demonstrated heterozygosity for the prothrombin G20210A mutation, methylenetetrahydrofolate reductase (MTHFR) polymorphisms, and reduced protein S activity. Because protein S activity may be influenced by acute thrombosis and vitamin K deficiency, repeat assessment after clinical stabilization was recommended.

## Discussion

This case represents the most severe end of the PE spectrum: recurrent PEA arrest, persistent shock, and marked right ventricular pressure overload. Acute pulmonary arterial obstruction abruptly increases right ventricular wall stress and oxygen demand; right ventricular failure then reduces left-ventricular preload and systemic perfusion, creating a self-perpetuating cycle of ischemia, hypotension, and arrest [1,4]. Rapid pulmonary reperfusion is therefore central to treatment.

Administration of systemic alteplase was consistent with the guideline-directed first-line reperfusion strategy for high-risk PE. In this patient, however, persistent vasopressor/inotrope-dependent cardiogenic-obstructive shock, electrical instability, and severe right ventricular overload indicated an inadequate early clinical response and justified urgent reassessment of the reperfusion strategy [1,3-5].

Evidence specifically addressing rescue thrombectomy after failed systemic thrombolysis is limited. The 2019 ESC/ERS guideline and the ESC Working Group/EAPCI consensus support consideration of catheter-directed treatment in high-risk PE when thrombolysis is contraindicated or has failed; the 2026 multisociety AHA/ACC guideline also considers mechanical thrombectomy reasonable in patients with persistent cardiogenic shock [1,3,4,6]. These recommendations largely reflect expert consensus and observational data rather than randomized trials.

The most directly relevant clinical series is a retrospective study of 48 patients treated with rescue catheter-based therapy after unsuccessful systemic thrombolysis. Oxygenation, shock index, RV/LV ratio, and pulmonary hypertension improved, with few reported procedural complications [7]. Nevertheless, the techniques were heterogeneous, failure was assessed later than in the present case, and the nonrandomized design introduces important selection and survivor biases.

The broader thrombectomy literature supports technical feasibility and rapid right ventricular unloading but cannot establish the benefit of rescue treatment after thrombolysis. The FLAME study prospectively evaluated mechanical thrombectomy in high-risk PE and reported favorable in-hospital outcomes relative to a prespecified performance goal, but it was nonrandomized and used a different device [8]. EXTRACT-PE and the STRIKE-PE interim analysis, using the Indigo aspiration platform, demonstrated prompt improvement in RV/LV ratio and acceptable short-term safety in predominantly intermediate-risk cohorts [9,10]. Thus, a mortality benefit, the ideal rescue device, and optimal timing after thrombolysis remain unproven.

Escalation approximately four hours after alteplase was driven by continued clinical instability. Bilateral aspiration was followed by a recorded pulmonary artery pressure decrease from 34 to 25 mmHg, but the patient remained in shock: the Day 1 clinical record documents severe instability despite vasoactive and inotropic support, and noradrenaline was still recorded on Day 6. Later circulatory recovery therefore cannot be presented as an immediate procedural endpoint. The separate contributions of alteplase, aspiration, anticoagulation, resuscitation, and ongoing intensive care cannot be determined from this single case.

The neurological course was documented by serial bedside assessments: on Day 6, without sedation, the patient grimaced in response to painful stimulation and had reactive pupils; by Day 23, she was awake and cooperative, and by Days 31-32, she was oriented and cooperative. Formal CPC and mRS scores cannot be retrieved from the clinical record. In a published case of high-risk PE complicated by out-of-hospital cardiac arrest, Thangavel et al. [11] described catheter-directed mechanical thrombectomy. This report provides a clinical comparison but does not establish the independent effect of thrombectomy in our patient.

The thrombophilia findings require cautious interpretation. Heterozygous prothrombin G20210A is a recognized venous thromboembolism risk factor, whereas common MTHFR polymorphisms do not independently establish thrombophilia or justify a change in management. Reduced protein S activity during acute thrombosis and critical illness should be confirmed after recovery and when assay-interfering treatments no longer affect the result [12,13].

This case report is limited by missing cumulative CPR duration, serial hemodynamic and laboratory measurements, complete preprocedural echocardiographic views, postprocedural echo images, and validated neurological outcome scores. Persistent shock after thrombectomy also prevents the pressure change from being presented as proof of immediate therapeutic success. Selection and temporal confounding preclude causal attribution or comparison with alternative rescue strategies.

## Conclusions

In this patient with high-risk PE and recurrent PEA arrest, alteplase was followed approximately four hours later by rescue bilateral aspiration thrombectomy because circulatory instability persisted. Pulmonary artery pressure decreased during aspiration, but shock resolved only gradually with ongoing intensive care. Reported biventricular function was normal at discharge. These observations support the feasibility of rescue aspiration in a selected patient; they do not establish its independent efficacy or optimal timing. Prospective comparative evidence and standardized serial outcomes remain necessary.

## References

[1] Konstantinides SV, Meyer G, Becattini C (2020). 2019 ESC Guidelines for the diagnosis and management of acute pulmonary embolism developed in collaboration with the European Respiratory Society (ERS). Eur Heart J.

[2] Kobayashi T, Pugliese S, Sethi SS (2024). Contemporary management and outcomes of patients with high-risk pulmonary embolism. J Am Coll Cardiol.

[3] Creager MA, Barnes GD, Giri J (2026). Guideline for the Evaluation and Management of Acute Pulmonary Embolism in Adults: a report of the American College of Cardiology/American Heart Association Joint Committee on Clinical Practice Guidelines. J Am Coll Cardiol.

[4] Pruszczyk P, Klok FA, Kucher N (2022). Percutaneous treatment options for acute pulmonary embolism: a clinical consensus statement by the ESC Working Group on Pulmonary Circulation and Right Ventricular Function and the European Association of Percutaneous Cardiovascular Interventions. EuroIntervention.

[5] Marti C, John G, Konstantinides S (2015). Systemic thrombolytic therapy for acute pulmonary embolism: a systematic review and meta-analysis. Eur Heart J.

[6] Giri J, Sista AK, Weinberg I (2019). Interventional therapies for acute pulmonary embolism: current status and principles for the development of novel evidence: a scientific statement from the American Heart Association. Circulation.

[7] Gong M, Chen G, Zhao B, Kong J, Gu J, He X (2021). Rescue catheter-based therapies for the treatment of acute massive pulmonary embolism after unsuccessful systemic thrombolysis. J Thromb Thrombolysis.

[8] Silver MJ, Gibson CM, Giri J (2023). Outcomes in high-risk pulmonary embolism patients undergoing FlowTriever mechanical thrombectomy or other contemporary therapies: results from the FLAME study. Circ Cardiovasc Interv.

[9] Sista AK, Horowitz JM, Tapson VF (2021). Indigo Aspiration System for treatment of pulmonary embolism: results of the EXTRACT-PE trial. JACC Cardiovasc Interv.

[10] Moriarty JM, Schiro BJ, Dohad SY (2025). Periprocedural results and right ventricular outcomes of computer-assisted vacuum thrombectomy treatment of acute pulmonary embolism: interim analysis of 300 patients from the STRIKE-PE study. J Am Heart Assoc.

[11] Thangavel S, Korsholm K, Veien KT, Larsen KM, Andersen A (2023). Catheter-directed mechanical thrombectomy in a patient with high-risk pulmonary embolism complicated by out-of-hospital cardiac arrest: a case report. Eur Heart J Case Rep.

[12] Arachchillage DJ, Mackillop L, Chandratheva A, Motawani J, MacCallum P, Laffan M (2022). Thrombophilia testing: a British Society for Haematology guideline. Br J Haematol.

[13] Hickey SE, Curry CJ, Toriello HV (2013). ACMG Practice Guideline: lack of evidence for MTHFR polymorphism testing. Genet Med.

